# COSGAP: COntainerized Statistical Genetics Analysis Pipelines

**DOI:** 10.1093/bioadv/vbae067

**Published:** 2024-05-09

**Authors:** Bayram Cevdet Akdeniz, Oleksandr Frei, Espen Hagen, Tahir Tekin Filiz, Sandeep Karthikeyan, Joëlle Pasman, Andreas Jangmo, Jacob Bergstedt, John R Shorter, Richard Zetterberg, Joeri Meijsen, Ida Elken Sønderby, Alfonso Buil, Martin Tesli, Yi Lu, Patrick Sullivan, Ole A Andreassen, Eivind Hovig

**Affiliations:** Department of Informatics, Centre for Bioinformatics, University of Oslo, Oslo 0373, Norway; Centre for Precision Psychiatry, Institute of Clinical Medicine, University of Oslo, Oslo 0450, Norway; Department of Informatics, Centre for Bioinformatics, University of Oslo, Oslo 0373, Norway; Centre for Precision Psychiatry, Institute of Clinical Medicine, University of Oslo, Oslo 0450, Norway; Centre for Precision Psychiatry, Institute of Clinical Medicine, University of Oslo, Oslo 0450, Norway; Centre for Precision Psychiatry, Institute of Clinical Medicine, University of Oslo, Oslo 0450, Norway; Centre for Precision Psychiatry, Institute of Clinical Medicine, University of Oslo, Oslo 0450, Norway; Department of Medical Epidemiology and Biostatistics, Karolinska Institutet, Stockholm 17177, Sweden; Department of Mental Health and Suicide, Norwegian Institute of Public Health, Oslo 0213, Norway; Unit of Integrative Epidemiology, Institute of Environmental Medicine, Karolinska Institutet, Stockholm 17177, Sweden; Institute of Biological Psychiatry, Mental Health Center Sct. Hans, Mental Health Services Copenhagen, Roskilde 4000, Denmark; The Lundbeck Foundation Initiative for Integrative Psychiatric Research (iPSYCH), Copenhagen 8210, Denmark; Institute of Biological Psychiatry, Mental Health Center Sct. Hans, Mental Health Services Copenhagen, Roskilde 4000, Denmark; The Lundbeck Foundation Initiative for Integrative Psychiatric Research (iPSYCH), Copenhagen 8210, Denmark; Institute of Biological Psychiatry, Mental Health Center Sct. Hans, Mental Health Services Copenhagen, Roskilde 4000, Denmark; The Lundbeck Foundation Initiative for Integrative Psychiatric Research (iPSYCH), Copenhagen 8210, Denmark; Centre for Precision Psychiatry, Institute of Clinical Medicine, University of Oslo, Oslo 0450, Norway; Department of Medical Genetics, Oslo University Hospital, Oslo 0424, Norway; Institute of Biological Psychiatry, Mental Health Center Sct. Hans, Mental Health Services Copenhagen, Roskilde 4000, Denmark; The Lundbeck Foundation Initiative for Integrative Psychiatric Research (iPSYCH), Copenhagen 8210, Denmark; Department of Mental Health and Suicide, Norwegian Institute of Public Health, Oslo 0213, Norway; Department of Medical Epidemiology and Biostatistics, Karolinska Institutet, Stockholm 17177, Sweden; Department of Medical Epidemiology and Biostatistics, Karolinska Institutet, Stockholm 17177, Sweden; Centre for Precision Psychiatry, Institute of Clinical Medicine, University of Oslo, Oslo 0450, Norway; KG Jebsen Centre for Neurodevelopmental Disorders, University of Oslo and Oslo University Hospital, Oslo 4956, Norway; Department of Informatics, Centre for Bioinformatics, University of Oslo, Oslo 0373, Norway; Department of Tumor Biology, Institute for Cancer Research, Oslo University Hospital, Oslo 0424, Norway

## Abstract

**Summary:**

The collection and analysis of sensitive data in large-scale consortia for statistical genetics is hampered by multiple challenges, due to their non-shareable nature. Time-consuming issues in installing software frequently arise due to different operating systems, software dependencies, and limited internet access. For federated analysis across sites, it can be challenging to resolve different problems, including format requirements, data wrangling, setting up analysis on high-performance computing (HPC) facilities, etc. Easier, more standardized, automated protocols and pipelines can be solutions to overcome these issues. We have developed one such solution for statistical genetic data analysis using software container technologies. This solution, named COSGAP: “COntainerized Statistical Genetics Analysis Pipelines,” consists of already established software tools placed into Singularity containers, alongside corresponding code and instructions on how to perform statistical genetic analyses, such as genome-wide association studies, polygenic scoring, LD score regression, Gaussian Mixture Models, and gene-set analysis. Using provided helper scripts written in Python, users can obtain auto-generated scripts to conduct the desired analysis either on HPC facilities or on a personal computer. COSGAP is actively being applied by users from different countries and projects to conduct genetic data analyses without spending much effort on software installation, converting data formats, and other technical requirements.

**Availability and implementation:**

COSGAP is freely available on GitHub (https://github.com/comorment/containers) under the GPLv3 license.

## 1 Introduction

Genome-wide association studies (GWAS) aim to determine the relationship between a phenotypic trait of interest and genetic data (Uffelmann *et al.* 2021). In the last decade, many GWAS approaches and corresponding tools have been developed for different applications such as PLINK (Purcell *et al.* 2007), GCTA (Yang *et al.* 2011), BOLT-LMM (Loh *et al.* 2015), SAIGE (Zhou *et al.* 2018), and REGENIE (Mbatchou *et al.* 2021). In addition, supplementary tools/packages for pre-processing or post-processing data are often required. Despite some common terminology and syntax, the tools have been created by different developers, and consequently, their required inputs in terms of data formats and flags are often different. Furthermore, native software installation on a machine can sometimes be challenging, due to technical issues related to the operating systems and software dependencies.

A challenge with GWAS and corresponding computational tools is consequently the user time spent to understand the parameters and options needed to operate them. Another challenge with sensitive data analysis is the difficulty of data sharing, e.g. due to the General Data Protection Regulation (GDPR). Sensitive genetic and phenotypic data are typically contained within secure facilities with controlled and limited internet access. Consequently, it is a challenge to provide the required packages/tools to the facilities to conduct the desired analysis. It becomes even more challenging to perform joint analyses of genetic data distributed across each partner facility. Since genetic data often cannot be shared among the sites, one straightforward solution is to apply the same tools, methodology, and computational pipeline to the data, and combine and/or compare the non-sensitive outputs, such as summary statistics, from these analyses.

There are existing pipelines aiming to solve some of the issues listed above. An automated GWAS pipeline called nf-GWAS (Song *et al.*, 2021), implemented in Nextflow and distributed with Docker containers, was developed using R-based tools, including SNPRelate/GENESIS/GMMAT and ANNOVAR. Another pipeline that uses Regenie for GWAS has been developed in (Schönherr *et al.* 2024). GWASpi, a JAVA-based platform to perform GWAS analysis using PLINK has been developed both as a web- and command-line-based platform (Muñiz-Fernandez *et al.* 2011). Comprehensive GWAS is another GWAS pipeline developed using the tools TASSEL and GAPIT (Dagasso *et al.*, 2020). The RICOPILI pipeline (Lam *et al.*, 2020) is a comprehensive pipeline that includes Quality Control (QC), imputation, and GWAS analysis. For post-GWAS analyses, such as Polygenic Scoring (PGS), there are also established pipelines such as GenoPred (Pain *et al.* 2021).

The use of software container technologies is well suited to overcome the challenges mentioned above in a scalable and sustainable fashion. Software containers can be built and distributed in multiple formats from one central location to the respective facilities, ensuring a consistent and replicable set of tools. They also add minimal computational overhead versus native execution of containerized tools (Alles *et al.*, 2018). Common container formats are Docker (https://docker.com), Singularity (https://docs.sylabs.io/), and Apptainer (https://apptainer.org), where the latter two are particularly suited for use on high-performance computing (HPC) facilities.

Built around a set of Linux-based Singularity containers supplemented by helper scripts and reference datasets, we here introduce COSGAP: “COntainerized Statistical Genetics Analysis Pipelines”. COSGAP is presently used across several genetic analysis projects. We also provide containerized Python and R installations with Jupyter and RStudio server support, respectively, with common dependencies for general scientific data analysis and visualization. Our approach does not only containerize existing tools in software containers, but also establishes a software distribution channel for users to download pre-built containers, with online documentation to conduct statistical genetic analysis. We provide many essential software for genotype data, QC, imputation, and statistical genetics analysis, including GWAS, PGS, and post-GWAS as listed in Table 1. In addition to these tools, we have also included relevant reference data with the available tools to ease the application by the end user. Furthermore, we included pipelines for GWAS analysis (PLINK and REGENIE), PGS analysis (PRSICE2 and LDpred2), and other statistical genetics analysis listed in Table 1. As shown in Fig. 2, we have standardized the GWAS pipeline so that users do not need to consider the variations in flags or data formats for different tools. This standardized pipeline includes a data specification procedure for genotype, phenotype, GWAS summary statistics data, and a Python-based command line user interface program. In addition to these standardized GWAS pipelines, we provide other tools for genetic data analysis with extensive documentation, showing both usage examples and best practices of statistical genetics analyses.

**Table 1.** List of analytical procedures and corresponding software tools.^a^

Container	Purpose:	Summary	Tools	Utilities
**hello.sif**	Demonstration	Demo container	PLINK1	
**gwas.sif**	GWAS	GWAS tools, meta-analyses	PLINK1, PLINK2, REGENIE, METAL, ++	gwas.py
**python3.sif**	*post-GWAS*	Post-GWAS and data analyses	Python, IPython, Jupyter server/notebook/lab, scipy-suite, common packages	
**r.sif**		Post-GWAS, polygenic scores, and data analyses	R, Rscript, Rserver, common packages, PRSice2, LDpred2	
**ldsc.sif**		LD score regression	LDSC	
**HDL.sif**		Genetic correlation estimation	HDL	
**MAGMA.sif**		Gene-set analysis, multi-trait local genetic correlation estimation,	MAGMA, LAVA, ldblock	
**MiXeR.sif**		Gaussian Mixture Models	Causal mixture models (MiXeR)	

a Note that these tools are developed by various groups. These have been listed in our GitHub repository and one should also cite these tools themselves, even if these tools are used with our pipeline.

One of the main assets of COSGAP compared to the pipelines mentioned above is its versatility both in terms of analysis and available tools. It enables conducting GWAS and PGS analyses similar to that of RICOPILI, but offers greater versatility in the choice of tools for a given analysis. COSGAP can perform GWAS with five different tools, including PLINK, GCTA’s fastGWA, SNPTEST, BOLT-LMM, and REGENIE, offering more flexibility. It is applicable to both case–control and quantitative traits, while RICOPILI is only designed for case–control studies.

COSGAP runs on both personal computers (PCs) and HPC facilities, and its main user interface is a command line program written in Python, gwas.py, plus user-editable YAML configuration files, that produce shell scripts for POSIX-compatible operating systems (e.g. Linux) for running the chosen analysis. The open-source project is actively developed, publicly available at github.com/comorment/containers (Frei *et al.* 2024; comorment/containers: Comorment-Containers-v1.8.1 (v1.8.1); Zenodo. https://doi.org/10.5281/zenodo.10782180), which is also used for distributing the project using Git (git-scm.com/) and the Git Large File System (LFS) extension (git-lfs.github.com), tracking of issues, and change-tracking of codes and files. Its documentation is hosted at cosgap.rtfd.io. The different software containers are based on the Ubuntu 20.04 (LTS) Linux distribution.

## 2 Methodology and technical requirements

**Figure 1.**
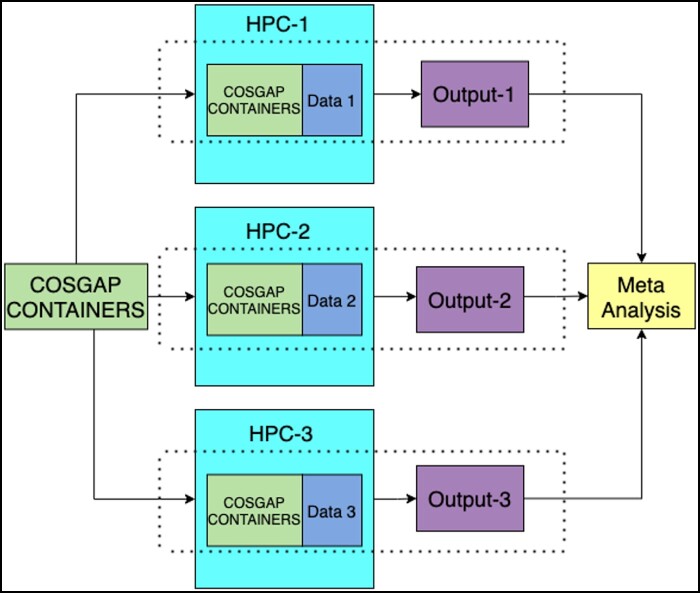
The diagram for distributed data analysis using COSGAP. COSGAP can be uploaded to each HPC system, allowing users to conduct distributed analysis.

COSGAP containers and analytical pipelines facilitate GWAS meta-analysis and leave-one-cohort-out PGS analysis, without individual-level data leaving each facility and can even run on systems without internet access. For security and privacy, our system relies on underlying two-factor authentication of the HPC platforms designed for handling sensitive human data. As depicted in Fig. 1, users at each site deploy COSGAP to their HPC system and perform their local analysis on their HPC system where sensitive genotype data are stored. The export of the non-sensitive output data is performed by following standard protocols for data exchange between systems, without involving our platform, and without additional data security requirements.

The proposed method can be regarded as secure, i.e. with regards to GDPR, as sensitive individual-level data never leaves each partner’s storage system. Also, code and containers are portable, and each site can upload and use the containers and other files directly. They are standardized, in the sense that each site may use the same embedded versions of software tools and corresponding files using a standardized pipeline. The amount of user input is minimalized in contrast to standard setups.

As stated before, our pipeline can be both used in HPC and PC. The technical requirements to run our pipeline are:

1. A POSIX-compatible host operating system (e.g. Unix, Linux, MacOS)
2. Singularity (https://sylabs.io/docs/) version 3 or higher, its community-driven version SingularityCE (https://sylabs.io/singularity/), or Apptainer (https://apptainer.org) on the host system.
3. A SLURM or Sun Grid Engine HPC scheduler

Only the first two requirements are necessary for running the pipeline on PC.

The software containers provide all relevant software in self-contained virtual machines that are built using the Ubuntu 20.04 (LTS) Linux-based operating system and rely only on the external dependencies of Singularity for running the containers, a Shell Command Language (sh) compatible shell (a Linux or Unix-like terminal application such as GNU Bash), and a job scheduler (e.g. SLURM) for submitting jobs to the HPC resource running the actual computing tasks. Some steps have also been made to ensure that the software environment within each container is consistent, i.e. Docker (docker.com) instruction files (Dockerfiles) and installation scripts request versions of each software that are explicitly defined during container rebuilds. Source codes and prebuilt containers are change-tracked using Git (https://git-scm.com) with Git LFS (https://git-lfs.github.com/), hosted publicly and freely under an open-source GPL-v3 license on GitHub, ensuring full transparency into their development history. GitHub is presently the main hub for issue tracking, coordinating the development, and running continuous integration tasks.

## 3 Application of the COSGAP pipeline

**Figure 2.**
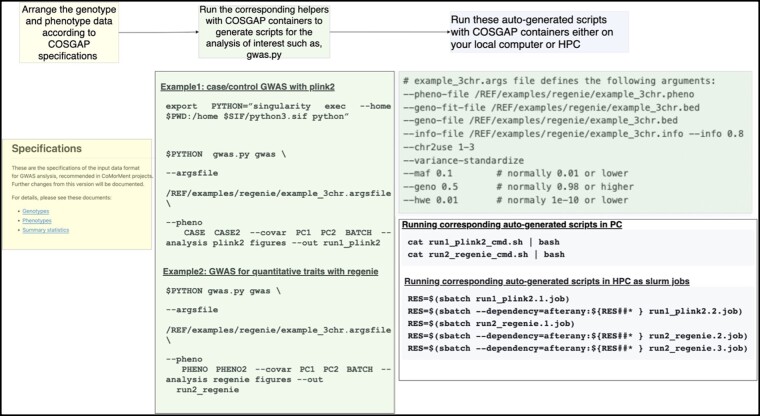
An illustrative example of the COSGAP pipeline for conducting GWAS analysis using different tools, such as PLINK and REGENIE. Initially, the data are arranged according to the specified format (https://cosgap.readthedocs.io/en/latest/specifications/README.html) and then run using gwas.py with the specified analysis (currently, it supports PLINK and REGENIE, for others you can use our documentation: https://cosgap.readthedocs.io/en/latest/usecases/README.html). Once gwas.py is run with desired options (in these examples, we run both PLINK and REGENIE with the figures option), scripts to run for PC and HPC are generated and the user can run these scripts without any modification and get the corresponding outputs with quantile–quantile and Manhattan plots.

This section defines how to install and use our COSGAP pipeline. A use case that shows how to conduct a demo GWAS analysis using COSGAP is presented in Fig. 2. At the time of writing, this manuscript corresponds to the release of version 1.9.0dev.

### 3.1 Installation

We recommend cloning the entire code repository (https://github.com/comorment/containers.git) using Git with the Git LFS extension enabled. The details of the installation can be found in the Installation section of our online docs. A minimal example is provided in the hello.sif container, which can be used to test that the pipeline works on the system.

### 3.2 Available tools

A complete, up-to-date list of tools available in COSGAP is available in the documentation. Some widely used tools are listed in Table 1. As can be seen from this list, various options for a given analysis (such as for GWAS, PGS) are available.

### 3.3 Workflow

COSGAP consists of containers and shared specifications for genotype, phenotype, and GWAS summary statistics files. To run different analyses, files must be restructured accordingly. Therefore, each workflow starts with data organization according to COSGAP specifications, as shown in Fig. 2. The next steps after data organization are running the containers with Python-implemented helper scripts (such as gwas.py) to generate scripts for the corresponding data analysis, and finally running these auto-generated scripts for conducting the analysis and getting the results. The steps for a GWAS analysis are shown in Fig. 2.

## 4 Discussion

We have here introduced the comprehensive and distributed genetic data analysis pipeline “COSGAP,” which relies extensively on software container technologies. Users can easily conduct GWAS and related analyses of their genetic data either locally or within secure HPC environments by using this pipeline. COSGAP incorporates a host of different tools, compared to other existing pipelines for statistical genetic data analyses. The number of included tools grows incrementally thanks to user-provided feedback. The solution allows researchers to use the most recent tools without spending significant time on software installation. In a federated analysis setting, it may also facilitate consistent versions of software and code used across individual sites and by the respective researchers.

In terms of usage, in addition to the statistical genetics analyses, different projects have already applied to the use of COSGAP containers for pre-imputation QC, phasing, imputation, and post-imputation, such as the MoBaPsychgen pipeline (Corfield *et al.*, 2024). It is also actively being used in several ongoing projects, especially from the Nordic countries, and has gained interest from other countries and projects. According to the feedback from the researchers using the pipeline, COSGAP will keep growing and remain up to date. Considering the versatility of our pipeline, we believe that COSGAP will also help researchers to conduct reproducible analysis more easily in various analysis problems.

Although COSGAP can be used both on local computers and HPC, support for Singularity containers is presently required to run COSGAP. However, support for Docker containers is considered and is feasible, as we presently rely on Docker to build Singularity container files themselves. The existing containers are also compatible with Apptainer (apptainer.org), a fork of Singularity.

Our aim is that COSGAP will remain a valuable tool for scientists to perform various genetic data analyses without spending time on technical details. Having such an automated genetic data analysis pipeline also enables standardization and this would make comparative and/or cooperative analyses easier.

## Data Availability

The data underlying this article are available as a GitHub repository (comorment/containers), at https://github.com/comorment/containers and also available at https://cosgap.readthedocs.io/en/latest/GETTING_STARTED.html.
